# Compliance with Iron and folic acid supplementation (IFAS) and associated factors among pregnant women: results from a cross-sectional study in Kiambu County, Kenya

**DOI:** 10.1186/s12889-018-5437-2

**Published:** 2018-05-02

**Authors:** Mary Wanjira Kamau, Waithira Mirie, Samuel Kimani

**Affiliations:** School of Nursing Sciences, College of Health Sciences, University of Nairobi, Nairobi, Kenya

**Keywords:** Compliance, Iron and folic acid supplementation, Pregnancy, Anaemia, Advice

## Abstract

**Background:**

Macro and micronutrients including iron and folic acid deficiencies are prevalent in Kenya, particularly during pregnancy resulting in anaemia. Despite efforts to control anaemia in pregnancy by adopting Iron and Folic Acid Supplementation (IFAS), this public health problem has persisted contributing to significant morbidity and mortality. The problem notwithstanding, there is poor IFAS compliance, whose reasons remain poorly understood, calling for their investigations. We sought to determine compliance status with IFAS and associated factors among pregnant women.

**Methods:**

This was a cross-sectional study involving 364 pregnant women aged 15–49 years. Using two stage cluster sampling, one Sub-County and five public health facilities in Kiambu County were selected. All pregnant women attending antenatal clinics who met inclusion criteria and consented to participate in the study were recruited. Compliance with IFAS was defined as taking supplements at least 5 out of 7 days per week. A structured interviewer-administered questionnaire consisting of sociodemographic data, IFAS maternal knowledge and compliance practices was pretested and administered. Descriptive and inferential statistics were computed using STATA.

**Results:**

Of the 364 respondents interviewed, 32.7% were IFAS compliant and 40.9% scored high on its knowledge. Of those with high IFAS knowledge, 48.3% were compliant compared to those with low knowledge (21.4%, *n* = 46, PR = 2.25;95%CI = 1.59–3.17, *p* < 0.001). Women who were multigravid (30.4%) were less likely to comply compared to primigravid (37.2%, *n* = 45, PR = 0.68;95%CI = 0.47–0.99, *p* = 0.004). Multivariate analysis revealed that respondents counselled on management of IFAS side effects (100%, *n* = 4) were more compliant (76.2%, *n* = 112, aPR = 1.31;95%CI = 1.19–1.44, *p* < 0.001).

**Conclusion:**

Few pregnant women were compliant with IFAS regimen, associated with: knowledgeability on IFAS, primi-gravidity, and IFAS counselling especially on management of its side effects. These underscore the need for approaches to scale up health awareness on the benefits of IFAS, mitigation measures for the side effects, as well as targeted counselling.

## Plain english summary

Lack of minute nutrients required in small amounts in the body is common in Kenya especially at important stages of life such as pregnancy. These can negatively affect growth and development of both mothers and their children. Lower than normal blood levels during pregnancy persist in Kenya contributing to death of one out of ten women and two out of ten infants. Despite provision of iron and folate supplements at no cost to all pregnant women in all government health facilities to help prevent these low blood levels, their use has been continually limited over the years. This study looked at consistency of women in using these daily supplements and factors influencing their use.

This study was conducted among pregnant women of child-bearing age in five public health facilities of one sub-County in Kiambu County, Kenya. A questionnaire was used to collect data from pregnant women.

Of all pregnant women interviewed, only a third were consistently using the supplements. Supplement use was higher among those advised on supplement use, those with high awareness of iron/folate supplements, those able to deal with side-effects and those pregnant for the first time.

### Conclusion

Consistency in iron/folate supplements use was low and positively influenced by receiving advice, ability to deal with side-effects, first-time pregnancy and having high awareness on iron/folate supplements. Gaps were identified in content of counselling emphasizing need to integrate other strategies like community based health education and focused counselling to increase supplement use.

## Background

Iron and folic acid are essential micronutrients for normal physiological function, growth and development as well as maintenance of life. Like many other nutrients, their demand increases during pregnancy creating a need for supplementation to meet daily requirement in pregnancy [1]. The high nutrient demand during pregnancy may not be met by regular diet because of insufficient amount and/or low bioavailability especially in the developing countries [2]. Due to this disproportion, deficiency in iron or folic acid causes an imbalance between demand and supply resulting in anaemia. Iron and folic acid and other micronutrient deficiencies are highly prevalent in Kenya, particularly at crucial stages of the life cycle such as pregnancy which can negatively impact the health of both mothers and children, before, during and after birth [3, 4].

Pregnancy-related anaemia remains top among the leading causes of global health burden [5], with more than half attributed to iron deficiency [6, 7], and the most common form of micronutrient malnutrition and prevalent nutritional deficiency leading to significant morbidity and mortality globally [7–10]. The global prevalence of pregnancy-related anaemia spans from 41.8 to 43.8% translating to approximately 59 million pregnant women [6], with the greatest (61.3%) burden in Africa, and South East Asia at 52.5% [11, 12]. Indeed, in developing countries, every second pregnant woman is estimated to be anaemic [7]. In Kenya, despite efforts to control and prevent anaemia in pregnancy, this public health problem persists, with a national prevalence of 55.1% [6, 9, 12], resulting in estimated 10% maternal and 20% perinatal deaths [13], respectively. The prevalence are similar to those found in Asia, where the problem is the second highest cause of maternal mortality [14]. Iron and folic acid supplementation (IFAS) during pregnancy has been recommended as one of the strategies to address this public health problem. It is an affordable and effective global strategy for prevention and control of anaemia during pregnancy resulting in reduced maternal-child morbidity and mortality [3, 13, 15].

Consistent with WHO guidelines [16], Kenya adopted IFAS programme as a high impact nutritional intervention to specifically address anaemia in pregnancy with a goal of achieving 80% coverage by 2017 [13]. IFAS is offered routinely to all antenatal mothers attending public health facilities, at no cost. Despite free distribution, compliance with IFAS has remained poor over the years with only about 8% pregnant women taking IFAS for more than 90 days, while over 30% do not take them at all [17–20]. Locally, at Thika hospital in Kiambu County, the County where this study was conducted, IFAS adherence rate (use of supplements for ≥4 days in a week) was shown to be 24.5% [21]. Similarly, in the neighbouring rural County of Machakos, the supplementation was found to be 18% [22] an indication that the problem is widespread.

One of the major reasons cited for poor IFAS response is poor compliance [15, 23], due to the requirement of daily supplementation [24]. Studies indicate that compliance with IFAS is generally poor, hindering IFAS success with subsequent poor maternal-child outcomes [15, 24, 25]. It has been found that those who need the supplements most, comply the least [26]. Many factors have been reported to affect IFAS compliance, notably health sector and client related issues, affecting and causing a lack of demand from both health sector and clients [27]. Specifically these factors include: forgetfulness, travel, age, literacy, socioeconomic status, cost of IFA tablets, perceived side effects, supplement stock-outs, birth order [28, 29], difficult accessing and poor utilization/frequency of antenatal health care services [30], and comprehensive knowledge of anaemia as well as quality of counselling on IFAS during pregnancy [29, 31, 32], including lack of clarity on importance of IFAS during pregnancy [32] due to inadequate counselling [29, 31, 33]. Other barriers include inadequate distribution of IFAS, beliefs against consuming medications during pregnancy, and fears that taking too much iron may cause too much blood or a big baby, making delivery difficult. It is noteworthy to mention that concern for maternal and fetal health positively influence taking IFAS [30] similar to improved maternal physical well-being and enhanced appetite with the alleviation of symptoms of anaemia, particularly fatigue [33]. Thus effectiveness and success of IFAS programme highly depends on the compliance with IFAS tablets.

Despite efforts to promote IFAS, there has not been commensurate decline in the number of women with anaemia or increased IFAS compliance in Kenya. There is need to scale up interventions to address this poor compliance. One of the critical measures taken by the Ministry of Health in addition to developing a lot of IFAS information education and communication (IEC) materials, has been to reduce the amount of iron per tablet from 200 mg to 60 mg since the frequency and severity of side effects increases with the dose of iron administered [34]. Further, combination of low dose iron and folic acid into one tablet was done to reduce pill burden thus increase compliance. Therefore, there is urgent need to address the factors affecting compliance and develop innovative strategies to mitigate them to increase IFAS coverage and eventually, substantially reduce the burden of pregnancy-related anaemia for improved maternal and child outcome. There is however, scarce information on the reasons for persistently low compliance with IFAS in Kenya. We sought therefore to determine the compliance status of pregnant women with IFAS and factors influencing their compliance.

## Methods

### Study site and design

This was a cross-sectional study conducted between June and October 2016 involving 364 pregnant women, aged 15–49 years, from Kiambu County, Kenya. One Sub-County and five of its public health facilities were sampled.

### Study population and sampling

Two-stage cluster sampling was used to identify one sub-county and five public health facilities. The two stages included selection of a sub-county and the health facilities. The sampling frame constituted of all the sub-counties in Kiambu County. The sub-county was selected because it had functional (active) community units, while the health facilities had high client/patient turnover. The target population were pregnant women aged 15–49 years living in Kiambu County.

### Sample size determination

The sample size was determined to establish prevalence of IFAS compliance, assuming the prevalence was 24.5% according to records obtained at Thika hospital, Kiambu County among women attending ANC clinic. The formula by Fisher 1999 [*n* = Z^2^pq/e^2^] for a cross sectional study was applied where n is the minimum sample score for a normal distribution (z = 1.96), p is the presumed prevalence of IFAS compliance (24.5%), q (1-p) is the proportion of non-compliance and e is the margin of error (e = 0.05). The minimum sample size was therefore 285. In addition, non-response rate of 30% was factored which resulted to a sample size of 370. The study achieved a sample size of 364 which is 98.5% response rate.

### Data collection methods

A structured, interviewer-administered questionnaire consisting of 33 closed-ended questions categorized into; socio-demographic data (12), maternal knowledge (9) and current practices towards IFAS (12), was developed, pretested and used in this study. To ensure reliability of the questionnaire, we adopted a test retest method where repeat pre-test was conducted after two weeks, while Cohen’s kappa statistic was used to measure the level of agreement of the results from the pre-tests. The questions which were tested and re-tested included: on socio-demographic data category: age, occupation, education level, income, parity, gravidity and gestation; on maternal knowledge: benefits of IFAS, possible side-effects of IFAS, how to manage the side effects, items on what happens if one does not get enough iron/folate, the signs and symptoms of anaemia, food sources that increase blood levels, how often should IFAS be taken, and how long should IFAS be taken; on current IFAS practices: number of IFAS tablets taken in the previous 7 days. Since all the questions repeated had a kappa value of above 0.7 after the comparison, the questionnaire was considered reliable, thus, all the questions were retained. To ensure validity, the tool was shared and discussed with experts from the Ministry of Health, division of nutrition, and the study supervisors. The obtained feedback was used to refine the tool. The questionnaires were administered by trained research assistants to all pregnant women who met the inclusion criteria and consented to the study. Pregnant women had no prior information before the interview regarding being assessed on compliance with IFAS because pre-testing had been carried out in a different sub-County.

### Data analysis

The outcome variable for this study was compliance with IFAS, assessed based on the reported number of IFAS tablets taken in the preceding 7 days before the interview. The IFAS compliance status was defined as the number of IFAS tablets taken in the preceding 7 days (1 week). Pregnant mothers who took at least 70% of the expected dose of the IFAS tablets in the week preceding the interview, an equivalent of five tablets per week, were considered compliant with IFAS [21, 35]. Conversely the respondents who took less than five IFAS tablets were considered as non-compliant. The compliance level was disaggregated to show the aforesaid categories of compliance. The independent variables included socio demographic characteristics (age, education and occupation of the pregnant mother), health care related factors (IFAS advice, parity, gravidity and gestation) and knowledge on IFAS. The respondents’ level of knowledge was computed by summing up all relevant 40 Likert scale items (5 items on benefits of IFAS, 7 on side-effects of IFAS, 6 on how to manage the side effects, 6 on what happens if you do not get enough IFAS, 7 on the signs and symptoms of anaemia, 7 on food sources that increase blood levels, an item on frequency of taking IFAS and an item on duration of taking IFAS). A correct answer for each item was scored as “1” and incorrect answer scored as “0”.

A generalized linear model with a Poisson distribution and a log link was used to account for clustering. This model was preferred over logistic regression as it generates more precise and accurate prevalence ratios and confidence levels. Results are reported as adjusted prevalence ratio (aPR) at 95% confidence interval (95%CI) with respective prevalence ratio used to assess the magnitude of the association where *p* < 0.05 denoted statistically significant results.

### Ethical considerations

The study received ethical approval from Kenyatta National hospital/University of Nairobi Ethics and Research Committee (KNH-UoN ERC/A/90 protocol number – P706/11/2015). Approval to conduct the study was also obtained from Kiambu County, Lari Sub-county authorities and all the health facilities involved. Participation in the study was purely voluntary. The study participants provided verbal and written informed consent before commencement of the interview. The minors (below 18 years of age) who agreed to participate in the study provided verbal and signed assent and their parents provided an informed consent on their behalf. The minors were included because in Kenya, the percentage of women who have begun childbearing increases rapidly with age, from about 3% among women age 15 to 40% among women age 19 years [17].

## Results

### Socio-demographic characteristics of study participants

A total of 364 pregnant women participated in the study. The range of respondents’ age was 16 to 47 years. The mean age of the study participants was 25.69 (SD ± 5.7), of which 68% were aged 19–29 years. Less than half (40.9%) had completed secondary education and beyond, while 39.3% had attained primary education. About half (52.5%) were employed with those earning USD 100 and above per month comprising only 7.4%. A majority (84.1%) were married, in their third trimester (76.9%), and multigravida (66.6%) (Table 1).

**Table 1:** Socio-demographic characteristics of respondents

	n	%
Age in years		
≤ 18	26	7.14
19–29	248	68.13
30 and Above	90	24.73
Occupation		
Employed	182	52.45
Unemployed	165	47.45
Level of education		
Primary	143	39.29
Secondary incomplete	72	19.78
Secondary complete	149	40.93
Average Income		
< 100 USD	325	92.59
≥ 100 USD	26	7.41
Number of children		
0	119	32.78
1	96	26.45
2	88	24.24
3+	60	16.53
Gravidity		
Primigravida	121	33.24
Multigravida	243	66.76
Gestation		
2nd trimester	84	23.08
3rd trimester	280	76.92
Mother’s IFAS knowledge		
Low knowledge	215	59.07
High knowledge	149	40.93

### Prevalence of IFAS compliance and associated factors

Of the 364 respondents, only 32.7% were IFAS compliant, while 40.9% scored high on the level of knowledge on IFAS. Those who scored above the median value were classified as having high IFAS knowledge, while those who scored below the median value were classified as having low IFAS knowledge. The distribution of the scores was approximately normal with mean 6.24 (SD = 3.64) and median 6.00. The mean score for IFAS knowledge among the compliant women was 7.71 (SD = 3.85) while that of the non-compliant respondents was 5.74 (SD = 3.21). None of the compliant respondents had anaemia in the first and second trimesters. In the third trimester, 50% (*n* = 1) of the respondents who had mild anaemia were compliant. During 1st and 2nd trimesters, 27.4% respondents were compliant compared to 34.3% during 3rd trimester. The compliance with IFAS was high among women who earned 100 USD or more (54%), were separated or divorced (50%), with high IFAS knowledge (49%) and aged 18 years and below (42%) (Table 2).

**Table 2:** Socio-demographic characteristics by compliance status among pregnant women

Socio-demographics	Compliant		Non-compliant			
	n	%(95%CI)	n	%(95%CI)	PR(95%CI)	*p*-value
Age in years						0.875
<=18	11	42.3(24.9–61.9)	15	57.7(38.1–75.1)	1.0	
19–29	79	31.9(26.2–37.8)	169	68.1(62.2–73.8)	0.75(0.44–1.28)	0.293
> 30	29	32.2(22.1–41.1)	61	67.8(58.9–77.9)	0.73(0.39–1.34)	0.309
Level of education						0.800
Primary	46	32.2(25.2–40.6)	97	67.8(59.4–74.8)	1.0	
Secondary incomplete	23	31.9(22.2–43.6)	49	68.1(56.4–77.8)	0.99(0.66–1.46)	0.945
secondary complete and above	50	33.6(25.2–40.2)	99	66.4(59.8–74.8)	0.99(0.71–1.39)	0.974
Occupation						0.764
Employed	59	32.4(25.4–39.1)	123	67.6(60.9–74.6)	1.07(0.79–1.43)	0.672
Unemployed	56	33.9(27.1–41.5)	109	66.1(58.5–72.9)	1.0	
Average Income						0.026*
< 100 USD	99	30.5(25.5–35.6)	226	69.5(64.4–74.5)	1.0	
≥ 100 USD	14	53.9(32.7–70.8)	12	46.2(29.2–67.3)	1.71(1.12–2.62)	0.013*
Number of children						0.127
0	46	38.7(30.3–47.8)	73	61.3(52.2–69.7)	1.0	
1	31	32.3(23.9–42.7)	65	67.7(57.3–76.1)	0.84(0.60–1.19)	0.332
2	24	27.3(18.2–36.8)	64	72.7(63.2–81.8)	0.68(0.47–0.99)	0.046*
3+	18	30.0(18.0–40.5)	42	70.0(59.5–82.0)	0.72(0.49–1.05	0.090
Gravidity						0.101
Primigravida	45	37.2(34.3–52.7)	76	62.8(47.3–65.7)	1.0	
Multigravida	74	30.4(23.8–35.4)	169	69.6(64.6–76.2)	0.68(0.52–0.88)	0.011*
Gestation						0.252
1st/2nd trimester	23	27.4(18.7–37.2)	61	72.6(62.8–81.3)	1.0	
3rd trimester	96	34.3(28.6–39.8)	184	65.7(60.2–71.4)	1.26(0.91–1.75)	0.171
Mother’s IFAS knowledge						0.003*
Low knowledge	46	21.4(16.5–27.5)	169	78.6(72.5–83.5)	1.0	
High knowledge	73	48.3(40.3–56.4)	76	51.7(43.6–59.7)	2.25(1.59–3.17)	< 0.001*

*PR* = Prevalence Ratio; *Significant at *P* < 0.05

### Analysis of factors associated with IFAS compliance

Results in Table 2 reveals that 27.3%; (*n* = 24) of the respondents with 2 children were less likely to be compliant compared to 38.7% (*n* = 46) of those with no children (PR = 0.68; 95%CI, 0.47–0.99; *P* = 0.046). Of the respondents who had some knowledge about IFAS, 48.3% (*n* = 73) were highly knowledgeable and were more compliant compared to those with low knowledge (21.4%, *n* = 46, PR = 2.25; 95%CI = 1.59–3.17, *p* < 0.001). Women who were multigravid (30.4%, *n* = 74) were less likely to be compliant compared to primigravid (37.2%, *n* = 45, PR = 0.68; 95%CI = 0.47–0.99, *p* = 0.004). Among the respondents who were compliant, 33.9% (*n* = 56) were unemployed compared to 32.4% (*n* = 59) employed. The difference was not statistically significant (PR = 1.07; 95%CI = 0.79–1.43, *p* = 0.772). Multivariate analysis conducted on the counselling status against IFAS compliance revealed that the respondents who were advised on the management of IFAS side effects (100%, *n* = 4) were more compliant than those who were not given this advice (76.2%, *n* = 112, aPR = 1.31; 95%CI = 1.19–1.44, *p* < 0.001) (Table 3).

**Table 3:** Multivariate regression analysis on IFAS compliance status among pregnant women

Advice content	Compliant		Non-compliant			
	N	%(95%CI)	n	%(95%CI)	aPR(95%CI)	*p*-value
Advice given						0.965
No	23	76.7(59.0–88.7)	7	23.3(11.3–42.0)	1.0	
Yes	93	76.9(68.4–83.6)	28	23.1(16.4–31.6)	1.00(0.80–1.26)	0.983
Side-effects						0.439
No	43	38.1(29.5–47.4)	70	61.9(52.6–70.5)	1.0	
Yes	52	43.7(35.0–52.8)	67	56.3(47.2–65.0)	1.15(0.84–1.58)	0.394
Management of the side effects						< 0.001*
No	112	76.2(68.6–82.4)	35	23.8(17.6–31.4)	1.0	
Yes	4	100.0(−)	0	0	1.31(1.19–1.44)	< 0.001*

*aPR* = Adjusted Prevalence Ratio; 95% CI =95% confidence intervals; *Significant at *P* < 0.05

### Practices and side-effects on IFAS

Among the compliant respondents who reported to have taken IFAS in the week prior the interview, the time of taking included: before meals (83%), before bedtime (81%), any time (80%) and in the morning (60%). However, the highest proportion (40%) among the non-compliant took the IFAS in the morning. Among the compliant respondents (77%), majority (95%) reported obtaining the tablets from a public health facility and the tablets lasted for mainly (82%) one month.

Challenges associated with taking IFAS included experiencing side-effects (12%), forgetting to take IFAS (4%), lack of adequate information (3%) and stock-outs (3%). The side-effects included nausea (23%) and epigastric pain (5%) which forced 22% of respondents to stop taking the tablets.

### Advice related to IFAS relative to compliance status

**Fig. 1:**
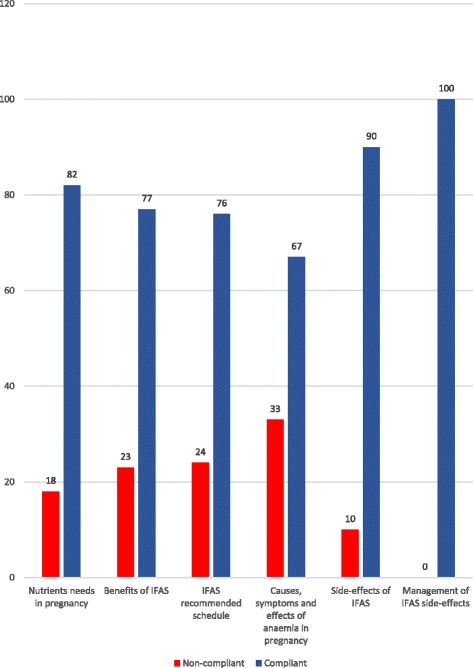
IFAS Advice given by compliance status. These refer to the categories of IFAS-related advice and counselling offered to the respondents during issuing of IFAS tablets at the antenatal clinic in relation to the compliance status of the respondents

Advice offered during issuing of IFAS at the health facility was commonly on their benefits (74%) and recommended schedule (22%). Compliance with IFAS varied according to advice given by health workers, being highest among those advised on side-effects and their management (Fig. 1). Of the respondents advised on the nutrients needs in pregnancy, 82.4% were compliant. Of those advised on the benefits of IFAS, 77% were compliant, while 76.5% were compliant with advice on the recommended schedule. The lowest proportion (66.7%) was among those advised on the causes, symptoms and effects of anaemia. Though only 6.54% were advised on the side effects of IFAS and 2.6% on their management, 100% (4) were compliant among those advised on management of IFAS side-effects.

## Discussion

Compliance with IFAS is found to be dismal at 32.7%. Similar findings have been reported in the neighbouring County of Machakos, Kenya (18%) [22] signifying a widespread problem. There is similarity of compliance pattern among the two counties despite their cultural and demographic diversity. These findings are consistent with reports on IFAS from similar rural settings in low and medium income countries (LMIC) notably, Pakistan (38.3%), South Ethiopia (39.2%) [35] and Western Ethiopia (20.4%) [36]. Despite efforts to promote IFAS, the finding could be attributed to the fact that compliance with medical advice depends on patients’ rational decision-making about the costs and benefits of prescribed actions, depending upon individual socio-cultural circumstances. In this regard, the key to improving compliance has been proposed through a suggestion to adopt a “more open, cooperative health professional-patient relationships” [11]. Open communication to clarify all issues is highly recommended. Factors associated with high compliance in this study are: (i) high knowledge on IFAS, (ii) advice regarding IFAS, (iii) ability to manage side effects, (iv) first time pregnancy, (v) high income and, (vi) age below 18 years. The study findings also revealed gaps in the content of IFAS advice in relation to IFAS side-effects and their management.

Knowledge on IFAS among pregnant women, coupled with awareness of anaemia and its relationship with IFAS are associated with improved compliance similar to reports from Ethiopia [35]. This may be related to the formal education where topics like anaemia are covered, as well as general exposure (experience) leading to better general understanding and health seeking behaviour. Promoting knowledge on anaemia improves compliance [29] just like the belief that IFAS increases blood level among women [37]. In addition, improved maternal physical well-being, alleviation of symptoms of anaemia, particularly fatigue, and enhanced appetite [33] have been shown to improve compliance with IFAS [30]. This underscores the critical role played by information, awareness creation and IFAS-related interventions. The IFAS policy implementation practices in Kenya should therefore be improved in relation to health education.

The IFAS advice given during the antenatal clinic was strongly implicated with promoting compliance. This is consistent with evidence where health education on IFAS accompanied by clear instructions on its intake improved compliance [35, 37]. Studies show increased awareness, appropriate counselling, effective communication, and health education on IFAS among pregnant women have tremendous improvement on its compliance [38, 39] necessitating targeted and focused information, training and counselling on importance of IFAS during pregnancy [28]. This leads to improved compliance and effective supplementation [40]. Thus, there is need to diversify strategies that deal with IFAS awareness creation both at health facility as well as community level to facilitate client education.

The ability to manage IFAS side-effects is associated with high compliance because side effects associated with IFAS significantly contribute to poor compliance and utilization [11, 29, 36, 41]. Despite IFAS side effects being a leading contributor to low compliance, most clients were not advised on their occurrence as well as their management. This made the women discontinue IFAS whenever they experienced side effects. It is therefore critical that clients are properly counselled on the possible side effects when IFAS tablets are issued as well as their management. Many health workers forget to mention IFAS side-effects to the clients thus contributing to poor or non-compliance following any slight discomfort [9]. All the women who were counselled on management of the side effects were found to be compliant. To minimize the side-effects, women are advised to eat plenty of fruits and vegetables and to take IFAS with meals or while going to bed. High doses of Vitamin C should also be avoided as it interferes with absorption of IFAS tablets. Ability to manage IFAS side effects will result in higher compliance. This is one of the IFAS topical areas that should be strengthened among health workers during IFAS counselling. Regular IFAS refresher courses among health workers are required to empower them. This will in turn empower pregnant women to manage side-effects whenever they occur rather than discontinuing IFAS which leads to non-compliance.

The first time pregnancy was associated with high IFAS compliance which is consistent with reports where primiparity was associated with high separate folic acid or iron tablets use [42]. This could be due to the fact that, a first time mother is very cautious and is therefore keen to follow advice on IFAS to ensure the best maternal and foetal outcome [30]. Women who have been pregnant before may also have experienced side effects before and were therefore hesitant to take IFAS tablets. They may take for granted advice during pregnancy especially if they do not experience any difficulties with the pregnancy. This shows the need to persistently teach pregnant women on importance of IFAS in their subsequent pregnancies. Strengthening peer counselling would help in this case.

The socio-economic factors notably high income and age below 18 years were associated with high IFAS compliance. Clients with high income are more likely to have attained high education level and hold formal employment, or own good businesses, all factors that favour compliance [35–37, 42, 43]. Level of education is a known determinant of formal employment and income [36, 44]. Other studies have shown that older age is associated with better IFAS use [35, 42]. This study found the opposite to be the case, which may be explained by the fact that younger women are fresh from school and are likely to have higher level of knowledge including on IFAS and anaemia. Most of the youngest women are also likely to be first time mothers hence keener on advice given. It is related to the fact that most first time pregnant women had higher compliance.

Our findings did not reveal major challenges experienced with IFAS programme. Although some studies cited side-effects as the major reason for stopping taking IFAS [11, 29, 36, 41], it was not a major challenge even though it was the highest reported challenge. There were other minor challenges that were reported which included, forgetting to take, lack of adequate information and stock-outs of IFAS. Taken together, the challenges could be surmounted through appropriate health education and targeted/focused counselling. Overall, the challenges could be mitigated using innovative approaches that target both the health sector and clients as the beneficiaries.

This study had a number of limitations namely: The 7 days’ compliance period is short and may not represent compliance throughout the pregnancy period. This period was utilized to minimize recall bias, being a cross-sectional study design, based on what previous studies had done [21, 28, 35]. Another limitation was recall bias and subjectivity because the study heavily relied on verbal reports from the interviewees. This challenge was circumvented through training of interviewers as well as double questioning to identify any inconsistencies in the interview reports. Fatigue due to lengthy and repeated interviews among pregnant women who are prone to mood swings and easy fatigability was mitigated through reassurance and psychosocial support to the respondents and correct timing for the interviews. Furthermore, participation to the study was purely voluntary. This ensured that their rights were respected and was in compliance with the ethical requirement for non-coercion. Finally, the study involved only one subcounty which may affect its generalizability to other counties. There is therefore need for further generalized studies with larger populations that take into account the entire pregnancy duration.

## Conclusion

Compliance with IFAS was found to be poor. Findings from this study reveal that factors that positively influenced IFAS compliance were: receiving advice on IFAS, high IFAS knowledge, counselling on management of IFAS side effects and first time pregnancy. It was also found that challenges experienced with IFAS were few and information-related such as gaps in content of IFAS counselling. These underscore the need to mitigate them through innovative approaches that strengthen health worker knowledge and capability to offer focused and targeted counselling so as to increase IFAS awareness among women and consequently compliance. This indicates necessity for improvement in IFAS policy implementation. We recommend introduction of diversified strategies in IFAS policy implementation like community based health education through community health workers to improve awareness on IFAS and monitor policy implementation as well as translation of knowledge into practice so as to consequently improve the compliance levels.

## Abbreviations

ERC: Ethics and Research Committee
IEC: Information, communication and education
IFAS: Iron and Folic Acid Supplementation
KDHS: Kenya National Demographic Health Survey
KNBS: Kenya National Bureau of Statistics
KNH: Kenyatta National Hospital
LMIC: Low and Medium Income Countries
MCSP: Maternal and Child Survival Program
MoH: Ministry of Health
MoPHS: Ministry of Public Health and Sanitation
UoN: University of Nairobi
WHO: World Health Organization
